# A Multi-Center Study on the Negative Psychological Impact and Associated Factors in Chinese Healthcare Workers 1 Year After the COVID-19 Initial Outbreak

**DOI:** 10.3389/ijph.2022.1604979

**Published:** 2022-08-25

**Authors:** Maria Jose Gonzalez Mendez, Li Ma, Ruben Alvarado, Jorge Ramirez, Kun-Peng Xu, Hui-Fang Xu, Shao-Kai Zhang, Mohamed S. Bangura, Ying Yang, Yan-Qin Yu, Xi Zhang, Wenjun Wang, Xiaofen Gu, Li Li, Didier Sama Salah, Youlin Qiao

**Affiliations:** ^1^ School of Public Health, Dalian Medical University, Dalian, China; ^2^ Department of Public Health, School of Medicine, Faculty of Medicine, Universidad de Valparaíso, Valparaíso, Chile; ^3^ School of Public Health, Faculty of Medicine, Universidad de Chile, Santiago, Chile; ^4^ Department of Quality Management, Dalian No. 3 People’s Hospital, Dalian, China; ^5^ The Affiliated Cancer Hospital of Zhengzhou University & Henan Cancer Hospital, Zhengzhou, China; ^6^ Department of Public Health and Preventive Medicine, Baotou Medical College, Baotou, China; ^7^ Key Laboratory of Carcinogenesis and Translational Research, Beijing Cancer Hospital, Peking University, Beijing, China; ^8^ Nursing School, Jining Medical University, Jining, China; ^9^ Department of Students Affairs, The Affiliated Cancer Hospital of Xinjiang Medical University, Urumqi, China; ^10^ Department of Clinical Research, First Affiliated Hospital of Jinan University, Guangzhou, China; ^11^ Department of Cardiology, First Affiliated Hospital of Dalian Medical University, Dalian, China; ^12^ Center for Global Health, School of Population Medicine and Public Health, Chinese Academy of Medical Sciences and Peking Union Medical College, Beijing, China

**Keywords:** mental health, healthcare workers, risk factors, COVID-19, psychological distress

## Abstract

**Objectives:** The study aimed at analyzing the prevalence of five psychological outcomes (depression, anxiety, stress, post-traumatic stress disorder (PTSD), and suicidal ideation) among Chinese healthcare workers (HCWs), and measured the total possible negative psychological impact 1 year after the COVID-19 initial outbreak.

**Methods:** A cross-sectional nationwide multi-center study was performed between November 2020 and March 2021 in China. A self-report questionnaire was applied, and three psychological scales were used. Binary logistic regression was performed to analyze the risk factors associated with each psychological outcome.

**Results:** The findings demonstrated that the COVID-19 pandemic had a negative psychological impact on HCWs, which was still evident 1 year after the initial outbreak. Nurses showed higher depression and anxiety than other HCWs. Female gender, passive coping, long working hours, having a chronic disease, and experiencing violence, among other factors, were all risk factors for psychological impairment.

**Conclusion:** Developing and promoting programs to improve mental health among HCWs, and identifying those who might need psychological support is still relevant 1 year after the initial outbreak.

## Introduction

The current COVID-19 pandemic has increased the levels of psychological distress in the general population [1] and other specific groups, such as students [2] and health professionals [3]. Healthcare workers (HCWs) played a primary role in containing the COVID-19 disease, including managing more patients at the facilities, long working hours, and being assigned to new work units [4]. These factors may affect the physical and psychological well-being of HCWs [5]. Even before the pandemic, studies reported that HCWs were more likely to develop burnout syndrome [6, 7] and high rates of anxiety and depression [8]. Evidence suggests that HCWs have suffered different levels of depression, anxiety, stress [9], post-traumatic stress disorder (PTSD) [10], insomnia [11], and suicidal ideation and attempts during the current pandemic [12]. Regarding the later, a higher risk of suicidal ideation and attempts have been observed throughout different phases of the pandemic, which might result from several factors such as COVID-19 related fears [13], uncertainty, and financial stressors [14]. In addition, previous pandemics have shown that HCWs can have high levels of PTSD manifest sometime after the critical period of the novel disease [15], which may manifest as a delayed response to a stressful event or situation [16].

China was the first country to deal with the new virus, firstly identified in Wuhan, Hubei province. Wuhan had the highest number of confirmed cases and deaths in China to date. At the critical stage of the pandemic, a considerable number of HCWs from all over the country were dispatched to Wuhan to collaborate with the local emergency. Studies have indicated that HCWs exposed to patients with COVID-19 reported depression, anxiety [17], and stress [18, 19]. Moreover, a high prevalence of PTSD has been found in Chinese HCWs 6 months after the initial COVID-19 outbreak [20].

Today, almost 2 years after the initial COVID-19 outbreak, the pandemic continues. In China, cases have significantly dropped since the beginning of 2020. According to information reported by WHO, as of 17 June 2022, China had a total of 4,127,625 cumulative COVID-19 cases. With the implementation of the COVID-Zero policy by the Chinese government, many regions have no COVID-19 cases, and the vaccination rate is one of the highest in the world, with more than 3 billion doses administered. However, the country’s preventive measures are still strict due to new waves of the disease. In November 2021, China experienced a new outbreak caused by the Delta variant. On 13 December 2021, China reported its first case of Omicron, which led to several containment measures such as canceling events, suspending business operations, and quarantining citizens from affected areas. The uncertainty of this ongoing period might maintain or increase adverse psychological outcomes among HCWs.

The present research aimed to screen five mental health problems: depression, anxiety, stress, PTSD, and suicidal ideation, and measure the overall negative psychological impact of the pandemic 1 year after the COVID-19 initial outbreak. In addition, we aimed to identify the main variables and risk factors associated with this negative psychological impact among Chinese HCWs from seven regions in China. We expect to provide scientific evidence for authorities to develop psychological interventions to improve the mental health status of HCWs in the current pandemic or future outbreaks.

## Methods

### Study Design and Participants

A cross-sectional multi-center study was conducted between 26 December 2020, and 29 March 2021. This study analyzed different health personnel, including doctors, nurses, laboratory technicians, mental health-related staff, Centers for Disease Control and Prevention (CDC)-related personnel, logistic personnel, administrative staff, and personnel of the medical technology department. Convenience sampling was utilized to invite potential participants to the study. The link to the questionnaire was forwarded by investigators to directors of local hospitals and CDC centers in the seven regions by direct message. Subsequently, they were in charge of forwarding the questionnaire link to health personnel in their respective facilities, which completed about 40% of the sample. HCWs from the different institutions in each region were asked to fill out the questionnaire and share it with other HCWs. To ensure the quality of the online survey, a control item was included—gender—which was asked twice in the questionnaire in different places as a basic information quality control.

**FIGURE 1:**
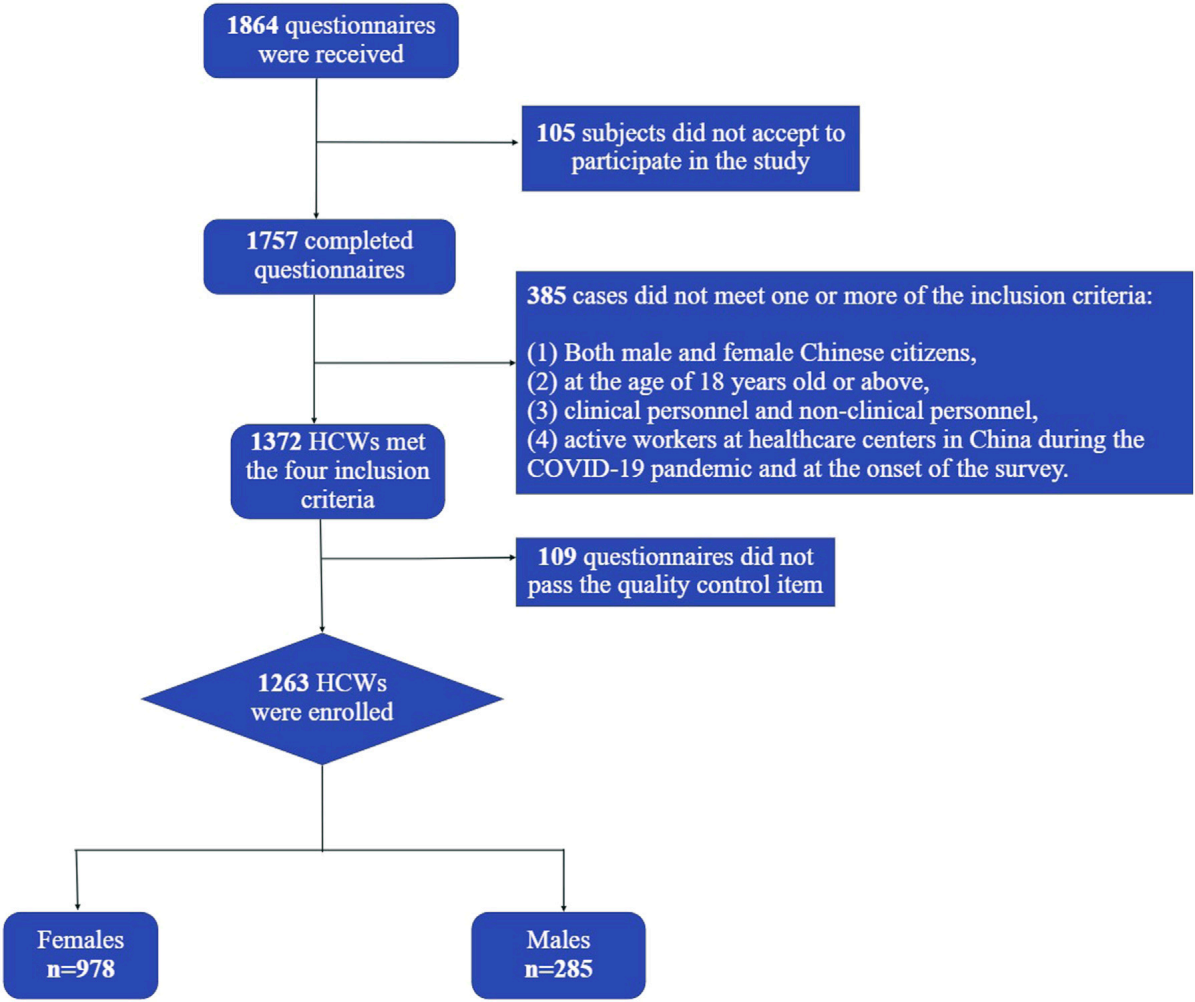
Recruitment flowchart (China, 2021).

Inclusion criteria for HCWs: 1) Both male and female Chinese citizens, 2) at the age of 18 years old or above, 3) clinical personnel and non-clinical personnel, 4) active workers at healthcare centers in China during the COVID-19 pandemic and at the onset of the survey. Respondents who were not HCWs, did not currently work at health facilities, and those that found difficulties using electronic devices were excluded. Figure 1 shows the flow chart of the recruitment process.

### Settings

The study participants were recruited from healthcare centers, including hospitals, public health institutions, primary care facilities, and CDC centers from seven Chinese regions, namely: the northwestern (NWC), northeastern (NEC), northern (NC), eastern (EC), southeastern (SEC), southern (SC), and central regions of China (CC). A local team of health professionals was formed in one of the main cities of each of the seven locations to recruit the study participants and collect the data per area.

### Ethics

This study and Informed Consent were approved by the Institutional Review Board of Dalian Medical University on 17 July of 2020 (2020007). Participation in the study was voluntary. The survey was displayed only if the subjects consented and agreed to participate in the study.

### Measurements

#### Sociodemographic Information

Sociodemographic data self-reported by the participants included: age, gender, occupation, education, parents’ education, members of the household, and previous health conditions.

#### Work Environment Information, Experiences, and Concerns During the COVID-19 Pandemic

The participants answered questions about their current work environments and experiences during the critical period of the pandemic.

### Depression, Anxiety, and Stress Scale-21

To assess depression, anxiety, and stress, we used the Chinese version of the Depression Anxiety and Stress Scale-21 (DASS-21). DASS-21 has been demonstrated to be valid for measuring psychological distress in the Chinese population, especially during the COVID-19 pandemic period [21–24]. DASS-21 includes three dimensions: depression, anxiety, and stress (seven items for each dimension). Cut-off scores of >9, >7, and >14 represented a positive screen of depression, anxiety, and stress. Participants were required to determine the presence of corresponding symptoms over the past week on a 4-point Likert scale ranging from 0 (did not apply to me at all) to 3 (applied to me very much or most of the time).

In this study, Cronbach alpha of the sub-scales of DASS-21 was 0.892, 0.884, and 0.903 for depression, anxiety, and stress, respectively.

### Primary Care PTSD Screen for DSM-5 (PC-PTSD-5)

The PC-PTSD-5 is a 5-item screening test designed to identify individuals with probable PTSD. It has demonstrated excellent diagnostic results in a study conducted on veteran primary care sample [25]. In the context of the COVID-19 pandemic, the Chinese version of the PC-PTSD-5 proved to be an efficient instrument to measure PTSD in medical staff exposed to the pandemic [26]. This scale is easy to apply and consists of five questions with a dichotomous (yes/no) response format, facilitating scoring and administration. In this study, the cut-off point used was 3, and the Cronbach alpha of the PC-PTSD-5 was 0.849.

### Patient Health Questionnaire-9

The PHQ-9 has been identified as the most reliable screening tool for depression and is widely validated in different countries [27] and China [28]. However, in this study, the PHQ-9 was not used to assess depression but suicidal ideation. To evaluate suicidal ideation, we used the last item of the PHQ-9 scale (have you ever hoped that you died or that you could fall asleep and never wake up again?). The last question of the PHQ-9 included in the questionnaire was dichotomous (Yes/No), and those respondents reporting “no” scored 0, while those reporting “‘yes” scored 1. This method has been previously used in a Chinese study measuring suicidal ideation during the COVID-19 pandemic [29].

### Simplified Coping Style Questionnaire (SCSQ-20)

Coping styles are thoughts and behaviors that people use to manage the internal and external demands of situations appraised as stressful [30]. Coping styles were assessed by the simplified coping style questionnaire (SCSQ) [31], which has been used in other studies during the COVID-19 pandemic in China [32, 33]. The SCSQ-20 is a 20 items scale with two dimensions: active/positive and passive/negative coping styles [34]. In this study, the Cronbach alpha was 0.937 and 0.842 for the positive and negative coping dimensions, respectively.

### Negative Psychological Impact

We computed a new variable called ‘negative psychological impact’ to calculate the overall psychological impact and avoid overlapping results (Supplementary Figure S1). To compute this variable, we used the results of each of the three psychological scales. When at least one of the five possible outcomes was present, or at least one point was obtained, we considered it as a negative psychological impact.

### Data Analysis

Statistical analysis was performed using IBM SPSS, version 24. The prevalence of mental health problems was derived according to the cut-off values suggested in previous studies in China. The internal consistency of the psychological scales was assessed using the Cronbach alpha coefficient. The frequency and proportion for count data in the questionnaire were calculated. The Chi-Square test was used to study the association between categorical variables. Fisher exact test was applied in case of small sample size. Kruskal-Wallis test was used to compare among groups. Binary logistic regression was used to analyze the factors associated with the risk of depression, anxiety, stress, PTSD, and suicidal ideation among the participants. We obtained a model for each psychological outcome using a stepwise backward elimination procedure. The associations between factors and outcomes were presented as odds ratios (ORs) and 95% confidence intervals. The statistical significance of all two-tailed tests was set at *p* < 0.05.

## Results

A total of 1,757 completed questionnaires were received; 385 did not meet the inclusion criteria of working at healthcare centers, and 109 did not pass the basic information quality control item. 1,263 participants were enrolled in this study (response rate: 71.8%). Table 1 presents sociodemographic characteristics of the whole sample and compares 396 doctors, 573 nurses, and 294 other health personnel (mental health-related staff, laboratory technicians, CDC-related staff, logistic personnel, administrative staff, and personal of the medical technology department). The average age of the sample was 35 ± 8.4 years, most participants were less than 35 years old (59%), and the majority were females (77.4%). Regarding previous conditions, only 0.3% of the participants reported having a psychological diagnosis prior to the pandemic, and 6.1% reported having a chronic disease diagnosis.

**TABLE 1:** Sociodemographic characteristics of HCWs by occupation (*n* = 1,263) (China, 2021).

Category	Total, *N* (%)	Doctors (*n* = 396)	Nurses (*n* = 573)	Other health personnel (*n* = 294)	*p*-value
Age (years)					**<0.001**
<35	756 (59.9)	162 (40.9)	416 (72.6)	178 (60.5)	
>35	507 (40.1)	234 (59.1)	157 (27.4)	116 (39.5)	
Gender					**<0.001**
Female	978 (77.4)	249 (62.9)	533 (93.0)	196 (66.7)	
Male	285 (22.6)	147 (37.1)	40 (7.0)	98 (33.8)	
Highest completed level of education					**<0.001**
Senior/high school/technical	33 (2.6)	6 (1.5)	19 (3.3)	8 (2.7)	
Junior high school	170 (13.5)	11 (2.8)	132 (23.0)	27 (9.2)	
Undergraduate degree	773 (61.2)	170 (42.9)	418 (72.9)	185 (62.9)	
Postgraduate	287 (22.7)	209 (52.8)	4 (0.7)	74 (25.2)	
Work population					**<0.001**
Children and adolescent	52 (4.1)	30 (7.6)	17 (3.0)	5 (1.7)	
Adults	484 (38.3)	150 (37.9)	251 (43.8)	83 (28.2)	
The elderly (over 65 years old)	135 (10.7)	37 (9.3)	85 (14.8)	13 (4.4)	
General population	540 (42.8)	169 (42.7)	202 (35.3)	169 (57.5)	
NA	52 (4.1)	10 (2.5)	18 (3.1)	24 (8.2)	
Type of medical unit you work at					**<0.001**
Primary medical and health institution	24 (1.9)	10 (2.5)	8 (1.4)	13 (3.3)	
Hospital	1,111 (88)	366 (92.4)	540 (94.2)	10 (1.7)	
Professional public health institution	90 (7.1)	7 (1.8)	15 (2.6)	15 (5.1)	
Other medical and health institution	38 (3.0)	13 (3.3)	10 (1.7)	38 (3.0)	
Live with anyone under 18 years old					**0.007**
Yes	736 (58.3)	244 (61.6)	346 (60.4)	736 (58.3)	
No	445 (35.2)	130 (32.8)	186 (32.5)	445 (35.2)	
N/R	82 (6.5)	22 (5.6)	41 (7.2)	82 (6.5)	
Live with anyone over 65 years old					0.107
Yes	246 (19.5)	89 (22.5)	114 (19.9)	43 (14.6)	
No	935 (74.0)	285 (72.0)	418 (72.9)	232 (78.9)	
N/R	82 (6.5)	22 (5.6)	41 (7.2)	19 (6.5)	
Previous mental health diagnosis					0.130^a^
No	91 (7.2)	25 (6.3)	51 (90.9)	15 (94.2)	
Yes	4 (0.3)	1 (0.3)	1 (0.2)	2 (0.7)	
I don’t want to answer	1,168 (92.5)	370 (93.4)	521 (90.9)	277 (94.2)	
**Previous chronic disease diagnosis**					**0.025**
No	1,123 (88.9)	353 (89.1)	519 (90.6)	251 (85.4)	
Yes	77 (6.1)	24 (6.1)	24 (6.1)	18 (6.1)	
I don’t want to answer	63 (5.0)	19 (4.8)	19 (3.3)	63 (5.0)	

a Fisher exact test used.

*p*-values in bold indicate statistical significance. Chi-square test used unless otherwise noted.

Only 2.5% of the respondents were sent to support Wuhan as a medical worker during the critical period of the pandemic, most of whom were nurses (4.2%). 77.4% of participants had close contact with suspected or confirmed COVID-19 cases. In total, 47.3% considered the PPE at their workplace insufficient and 5.3% very insufficient; among the three groups, more doctors considered the PPE insufficient or very insufficient. 13.1% were very worried about infecting relatives. 8.3% of participants felt stigmatized or discriminated by the society in their role as HCW (by patients, relatives, etc.) during the outbreak. In addition, 10.6% of the respondents experienced violence during the pandemic. Details of the experiences during the pandemic and differences among health personnel are shown in Table 2.

**TABLE 2:** Experiences, fears, concerns, and work environment during the COVID-19 pandemic (China, 2021).

Category	Total, *N* (%)	Doctors (*n* = 396)	Nurses (*n* = 573)	Other health personnel (*n* = 294)	*p*-value
Assigned to a new team or functions					**0.002**
Yes	569 (45.1)	150 (37.9)	282 (49.2)	137 (46.6)	
No	694 (54.9)	246 (62.1)	291 (50.8)	157 (53.4)	
Working days in a week during the outbreak					**<0.001**
≤5	695 (55.0)	184 (46.5)	357 (62.3)	154 (52.4)	
>5	568 (45.0)	212 (53.5)	216 (37.7)	140 (47.6)	
Daily working hours					**0.042**
1 to 8	749 (59.3)	215 (54.3)	357 (62.3)	177 (60.2)	
9 to 24	514 (40.7)	181 (45.7)	216 (37.7)	117 (39.8)	
Place of work during the outbreak					**0.001**
Work unit	1,058 (83.8)	344 (86.9)	486 (84.8)	228 (77.6)	
At home	39 (3.1)	10 (2.5)	9 (1.6)	20 (6.8)	
Both (work unit and at home)	139 (11.0)	35 (8.8)	64 (11.2)	40 (13.6)	
Other	27 (2.1)	7 (1.8)	14 (2.4)	6 (2.0)	
Close contact with patients who were suspected or confirmed COVID-19 cases					
Yes	978 (77.4)	330 (83.3)	432 (75.4)	216 (73.5)	**0.009**
No	226 (17.9)	50 (12.6)	116 (20.2)	60 (20.4)	
I don’t know	59 (4.7)	16 (4.0)	25 (4.4)	18 (6.1)	
Went to Wuhan during the outbreak					0.099
Yes	39 (3.1)	10 (2.5)	24 (4.2)	5 (1.7)	
No	1,224 (96.9)	386 (97.5)	549 (95.8)	289 (98.3)	
Reason to go to Wuhan					**0.044** ^a^
Support as a medical worker	32 (2.5)	9 (2.3)	21 (3.7)	2 (0.7)	
Visit friends/relatives	3 (0.2)	1 (0.3)	1 (0.2)	1 (0.3)	
Other	4 (0.3)	0 (0.0)	2 (0.3)	0 (0.0)	
N/R	1,224 (96.9)	386 (97.5)	549 (95.8)	289 (98.3)	
PPE availability					**<0.001**
Very insufficient	67 (5.3)	33 (8.3)	26 (4.5)	8 (2.7)	
Not enough	598 (47.3)	211 (53.3)	256 (44.7)	131 (44.6)	
Enough	592 (46.9)	149 (37.6)	288 (50.3)	155 (52.7)	
Not applicable	6 (0.5)	3 (0.8)	3 (0.5)	0 (0.0)	
Worried about getting COVID-19					**0.001**
Not worried at all	132 (10.5)	40 (10.1)	57 (9.9)	35 (11.9)	
A little worried	773 (61.2)	252 (63.6)	322 (56.2)	199 (67.7)	
Quite worried	193 (15.3)	64 (16.2)	95 (16.6)	34 (11.6)	
Very worried	165 (13.1)	40 (10.1)	99 (17.3)	26 (8.8)	
Days in isolation for being suspected or a confirmed case of COVID-19					**<0.001**
0	978 (77.4)	328 (82.8)	409 (71.4)	241 (82.0)	
1 to 15	230 (18.2)	48 (12.1)	135 (23.6)	47 (16.0)	
16 to 30	29 (2.3)	10 (2.5)	14 (2.4)	5 (1.7)	
More than 30	26 (2.1)	10 (2.5)	15 (2.6)	1 (0.3)	
Worried about infecting your relatives					**<0.001**
Not worried at all	39 (3.1)	10 (2.5)	16 (2.8)	13 (4.4)	
A little worried	430 (34.0)	152 (38.4)	160 (27.9)	118 (40.1)	
Quite worried	368 (29.1)	133 (33.6)	148 (25.8)	87 (29.6)	
Very worried	426 (33.7)	101 (25.5)	249 (43.5)	76 (25.9)	
As a health worker, I feel stigmatized or discriminated due to the new COVID-19 epidemic					0.294	
Strongly disagree	614 (48.6)	184 (46.5)	274 (47.8)	156 (53.1)	
Disagree	543 (43.0)	170 (42.9)	254 (44.3)	36 (6.3)	
Agree	89 (7.0)	37 (9.3)	36 (6.3)	16 (5.4)	
Strongly agree	17 (1.3)	5 (1.3)	9 (1.6)	3 (1.0)	
As a health worker, I have experienced violence during the pandemic					**0.007**
Strongly disagree	551 (43.6)	154 (38.9)	246 (42.9)	151 (51.4)	
Disagree	578 (45.8)	201 (50.8)	254 (44.3)	123 (41.8)	
Agree	119 (9.4)	36 (9.1)	67 (11.7)	16 (5.4)	
Strongly agree	15 (1.2)	5 (1.3)	6 (1.0)	4 (1.4)	
Patients with COVID-19 that you directly cared for passed away					**0.009**
No	1,143 (91.1)	353 (89.6)	512 (90.3)	278 (94.6)	
Yes	79 (6.3)	27 (6.9)	45 (7.9)	7 (2.4)	
I don’t know	33 (2.6)	10 (1.8)	10 (1.8)	9 (3.1)	
I have a reliable social support at work					0.550
Disagree	121 (9.6)	43 (10.9)	50 (8.7)	28 (9.5)	
Agree	1,142 (90.4)	353 (89.1)	523 (91.3)	266 (90.5)	
The pandemic affected the family’s financial situation					**<0.001**
Yes	845 (66.9)	268 (67.7)	409 (71.4)	168 (57.1)	
No	418 (33.1)	128 (32.3)	164 (28.6)	126 (42.9)	

a Fisher exact test used.

*p*-values in bold indicate statistical significance.

Chi-square test used unless otherwise noted.

According to standard questionnaire cut-offs of the DASS-21, 26.7%, 33.4%, and 14.8% of the participants presented with more probability of depression, anxiety, and stress, respectively. Similarly, the PC-PTSD-5 scale showed 10.1% of the participants with positive symptoms of PTSD, and 8.3% of the participants had suicidal thoughts in the last month. Regarding the negative psychological impact, of 1,263 participants, 549 presented with at least one mental health problem 1 year after the COVID-19 initial outbreak. No significant differences were observed in the prevalence of any mental health outcome among different health personnel (Table 3). However, nurses had higher total scores in the DASS-21 depression (*p* = 0.016) and stress (*p* = 0.011) sub-scales. Similarly, nurses showed significantly higher scores in certain specific items of the DASS-21 scale, such as having difficulties taking the initiative at work (*p* = 0.004), feeling depressed (*p* = 0.020), unable to become enthusiastic (*p* = 0.030), feeling about to collapse (*p* = 0.021), finding it difficult to calm down (*p* = 0.020), feeling like I consume a lot of energy (*p* = 0.014), and finding it hard to relax (*p* = 0.003). The total scores of the anxiety sub-scale and PC-PTSD-5 showed no significant differences among occupations (Table 4).

**TABLE 3:** Prevalence of mental health problems in the total sample and by occupation (China, 2021).

Variable	Total, *N* (%)	Doctors (*n* = 396)	Nurses (*n* = 573)	Other health personnel (*n* = 294)	*p*-value^a^
Depression					0.902
Yes	338 (26.8)	107 (27.0)	150 (26.2)	81 (27.6)	
No	925 (73.2)	289 (73.0)	423 (73.8)	213 (72.4)	
Anxiety					0.204
Yes	422 (33.4)	120 (30.3)	205 (35.8)	97 (33.0)	
No	841 (66.6)	276 (69.7)	368 (64.2)	197 (67.0)	
Stress					0.331
Yes	186 (14.7)	57 (14.4)	78 (13.6)	51 (17.3)	
No	1,077 (85.3)	339 (85.6)	495 (86.4)	243 (82.7)	
PTSD					0.267
Yes	128 (10.1)	33 (8.3)	66 (11.5)	29 (9.9)	
No	1,135 (89.9)	363 (91.7)	507 (88.5)	265 (90.1)	
Suicidal ideation					0.147
Yes	105 (8.3)	29 (7.3)	57 (9.9)	19 (6.5)	
No	1,158 (91.7)	367 (92.7)	516 (90.1)	275 (93.5)	
Negative psychological impact (at least one mental health problem)					0.396
Yes	549 (43.5)	165 (41.7)	261 (45.5)	123 (41.8)	
No	714 (56.6)	231 (58.3)	312 (54.5)	171 (58.2)	

a Chi-square test used unless otherwise noted.

**TABLE 4:** Psychological manifestations among doctors, nurses and other health personnel (China, 2021).

Characteristics	Total (*n* = 1,263)	Doctor (*n* = 396)	Nurse (*n* = 573)	Other health personnel (*n* = 294)	*p*-value^a^
Total DASS-21 depression sub-scale score	5.85 ± 6.99	5.44 ± 6.54	6.49 ± 7.48	5.17 ± 6.49	**0.016**
Don’t feel any pleasure or comfort at all	0.60 ± 0.72	0.55 ± 0.66	0.65 ± 0.77	0.57 ± 0.69	0.228
Difficulties taking the initiative at work	0.46 ± 0.67	0.40 ± 0.60	0.54 ± 0.72	0.40 ± 0.63	**0.004**
I feel that I have nothing to look forward to in the near future	0.50 ± 0.71	0.47 ± 0.70	0.55 ± 0.75	0.43 ± 0.65	0.071
I feel depressed	0.52 ± 0.68	0.49 ± 0.65	0.57 ± 0.70	0.45 ± 0.66	**0.020**
Unable to become enthusiastic	0.45 ± 0.67	0.44 ± 0.64	0.50 ± 0.70	0.37 ± 0.62	**0.030**
I feel I am not worth much	0.15 ± 0.45	0.16 ± 0.45	0.16 ± 0.48	0.13 ± 0.39	0.776
I feel life is meaningless	0.24 ± 0.55	0.20 ± 0.48	0.28 ± 0.61	0.22 ± 0.54	0.155
Total DASS-21 anxiety sub-scale score	6.15 ± 7.04	5.70 ± 6.48	6.66 ± 7.62	5.76 ± 6.51	0.169
I feel dry mouth	0.66 ± 0.75	0.62 ± 0.69	0.69 ± 0.78	0.66 ± 0.76	0.496
I have difficulty breathing (wheezing or breathless)	0.36 ± 0.61	0.37 ± 0.57	0.39 ± 0.65	0.30 ± 0.55	0.105
I feel trembling (for example, shaking hands)	0.28 ± 0.57	0.27 ± 0.54	0.30 ± 0.61	0.25 ± 0.51	0.760
I worry about occasions that might make me panic or make a fool of myself	0.68 ± 0.73	0.62 ± 0.67	0.71 ± 0.75	0.68 ± 0.75	0.333
I feel about to collapse	0.38 ± 0.64	0.33 ± 0.60	0.44 ± 0.70	0.33 ± 0.56	**0.021**
Even when there is no obvious physical activity, I feel my heart rhythm is abnormal	0.38 ± 0.66	0.34 ± 0.61	0.42 ± 0.72	0.35 ± 0.61	0.469
I feel scared for no reason	0.33 ± 0.62	0.30 ± 0.55	0.37 ± 0.67	0.31 ± 0.59	0.383
Total DASS-21 stress sub-scale score	7.77 ± 7.91	7.04 ± 7.26	8.65 ± 8.55	7.05 ± 7.29	**0.011**
I find it difficult to calm myself down	0.56 ± 0.68	0.48 ± 0.60	0.64 ± 0.75	0.53 ± 0.61	**0.020**
I tend to over-react	0.40 ± 0.64	0.37 ± 0.60	0.45 ± 0.68	0.36 ± 0.61	0.070
I feel that I am using a lot of energy	0.73 ± 0.80	0.64 ± 0.70	0.82 ± 0.87	0.69 ± 0.75	**0.014**
I feel uneasy	0.48 ± 0.66	0.45 ± 0.61	0.52 ± 0.70	0.45 ± 0.65	0.285
I find it hard to relax	0.60 ± 0.74	0.57 ± 0.70	0.68 ± 0.79	0.49 ± 0.68	**0.003**
I can’t tolerate anything that prevents me from continuing to work	0.55 ± 0.73	0.50 ± 0.68	0.60 ± 0.76	0.51 ± 0.71	0.080
I found that I was easily offended	0.56 ± 0.72	0.52 ± 0.70	0.62 ± 0.77	0.50 ± 0.64	0.066
Total PC-PTSD-5 scale (last 6 months) score	0.59 ± 1.27	0.53 ± 1.21	0.66 ± 1.35	0.56 ± 1.19	0.352
Often have nightmares or always thinking about COVID-19 inadvertently	0.13 ± 0.34	0.11 ± 0.31	0.14 ± 0.35	0.12 ± 0.33	0.238
Often try to avoid thinking of the COVID-19 pandemic, or try to avoid scenarios that remind you of it	0.13 ± 0.34	0.12 ± 0.32	0.14 ± 0.35	0.12 ± 0.32	0.394
Had been on alert, vigilant or often easily frightened	0.15 ± 0.35	0.13 ± 0.34	0.15 ± 0.36	0.15 ± 0.35	0.693
Had often felt numb or out of touch with people, activities or surroundings	0.12 ± 0.33	0.11 ± 0.32	0.13 ± 0.34	0.08 ± 0.32	0.562
Feel guilty or often blame yourself for COVID-19 and related things	0.07 ± 0.25	0.05 ± 0.22	0.08 ± 0.28	0.06 ± 0.24	0.184
PHQ-9 item 9					
Have you ever hoped that you died, or that could fall asleep and never wake up again?	0.08 ± 0.276	0.06 ± 0.23	0.10 ± 0.30	0.09 ± 0.28	0.412

*p*-values in bold indicate significant differences among groups.

a Obtained from the comparison among occupations using the Kruskal Wallis H Test.

Multivariate analysis showed that two of the analyzed factors—using a passive coping style and long working hours during the outbreak—increased the risk of each of the five psychological outcomes. Furthermore, experiencing violence as a healthcare worker during the pandemic, having prior chronic diseases, and close contact with COVID-19 confirmed or suspected cases were associated with a higher risk of four psychological outcomes. On the other hand, factors such as having reliable social support at work, not being a doctor or a nurse, and older age were observed as protective factors for some of the analyzed outcomes. The summary of the significant variables associated with each outcome is displayed in Table 5.

**TABLE 5:** Summary of the risk and protective factors for depression, anxiety, stress, PTSD, and suicidal ideation (China, 2021).

Variable	Depression	Anxiety	Stress	PTSD	Suicidal ideation
	OR [95% CI]	OR [95% CI]	OR [95% CI]	OR [95% CI]	OR [95% CI]
Older age	—	0.96 [0.95–0.98]	—	—	0.97 [0.94–1.00]
Female gender	1.55 [1.07–2.24]	1.36 [0.97–1.91]	—	—	2.95 [1.49–5.86]
Previous chronic disease	2.06 [1.24–3.43]	2.13 [1.28–3.53]	2.70 [1.55–4.70]	—	3.04 [1.56–5.92]
Experienced violence during the pandemic	1.86 [1.25–2.76]	2.09 [1.41–3.10]	2.37 [1.52–3.67]	1.94 [1.18–3.20]	—
Passive coping	3.00 [2.25–4.01]	3.05 [2.34–3.97]	2.42 [1.68–3.47]	2.35 [1.55–3.56]	3.26 [1.96–5.42]
Having reliable social support at work	0.45 [0.30–0.67]	—	0.46 [0.29–0.72]	—	0.49 [0.29–0.85]
Long working hours during the outbreak	1.11 [1.06–1.17]	1.07 [1.02–1.12]	1.13 [1.07–1.19]	1.07 [1.01–1.14]	1.13 [1.06–1.20]
More days in isolation during the pandemic	—	—	1.03 [1.01–1.04]	1.04 [1.01–1.14]	—
Other health personnel	0.56 [0.37–0.84]	0.48 [0.33–0.70]	—		—
Aided Wuhan during the pandemic	2.08 [1.02–4.26]	2.30 [1.12–4.75]	—	—	—
Quite worried or very worried about infecting relatives	—	1.37 [1.04–1.79]	—	—	—
The pandemic affected the family’s financial situation	1.38 [1.02–1.85]	–	1.50 [1.04–2.17]	—	—
Close contact with COVID-19 patients	2.89 [1.77–4.74]	2.83 [1.79–4.46]	1.95 [1.11–3.46]	3.68 [1.54–8.80]	—

## Discussion

The findings on the prevalence of mental health problems among HCWs 1 year after the initial COVID-19 outbreak are a point of concern. This study analyzed the probable prevalence of depression, anxiety, stress, PTSD, and suicidal ideation and found that 1 year after the initial COVID-19 outbreak, 43.5% of the participants manifested symptoms of at least one of the five analyzed psychological outcomes. In addition, the analysis of each psychological outcome showed 33.4% of anxiety, 26.8% of depression, 14.7% of stress, 10.1% of PTSD symptoms, and 8.3% of suicidal thoughts among the participants.

Additionally, this study analyzed different types of health personnel. We observed that nurses had higher scores on the scales of depression and stress than doctors and other health personnel. Other health personnel (like administrative staff, laboratory technicians, etc.) had a lower risk of developing symptoms of depression and anxiety, which is consistent with previous studies during the pandemic [35, 36]. Nurses play a primary role in the response to COVID-19 and, therefore, have been more exposed to emotional exhaustion [37] since they are directly involved in patients’ treatments [38]. This might cause their higher rates of symptoms of depression and stress.

Our findings are consistent with previous studies performed during the pandemic reporting high rates of mental health problems among HCWs [39]. However, our study’s prevalence of depression, anxiety, and stress was lower than those obtained in a Chinese study during the critical pandemic period that showed 37.8% of depression, 43.0% of anxiety, and 38.5% of stress among HCWs from different provinces [40]. This difference might be related to when the mentioned study was performed, because the level of uncertainty about the virus was higher at the beginning of the outbreak. In addition, the mentioned study included Wuhan, the starting point of the epidemic in China, which may be related to higher rates of psychological unrest. Moreover, a similar study performed 1 year after the initial outbreak found depression, anxiety, and PTSD levels of 49%, 38%, and 56% in a control group of HCWs that did not work in Wuhan [41]. Although the percentages of that study are higher than ours, it corroborates the presence of psychological problems in HCWs 1 year after the initial COVID-19 outbreak. Though China announced that the community transmission had been controlled since the middle of 2020 [42] and cases have significantly dropped, the preventive measures are still strict in the country due to new waves of the disease, which might be related with the high rates of psychological symptoms 1 year after the initial outbreak.

Our findings reported considerable PTSD symptoms among participants, consistent with similar studies conducted in China six and 7 months after the initial lockdown, that found 10.5% and 4.8% of PTSD symptoms among HCWs [43, 44], respectively. Our work demonstrated that the risk of manifesting PTSD symptoms increased in participants that: used passive coping strategies, experienced violence during the pandemic, worked long daily hours during the outbreak, were isolated for more days, and had close contact with COVID-19 cases. Most of the mentioned factors are closely related to the pandemic, and should be considered in future outbreaks or health emergencies to avoid adverse psychological outcomes in HCWs.

Regarding suicidal ideation, our results are consistent with those found in a large-scale study performed during the critical period of the COVID-19 pandemic in China that reported 6.47% of suicidal ideation and self-harm among HCWs [45]. Similarly, studies in other countries have shown a prevalence of suicidal ideation ranging from 3.6% to 8.4% during the COVID-19 pandemic [46]. Previous studies showed that chronic illness might induce depression and suicidal ideation [47, 48], which is consistent with the findings of our study that identified having a previous chronic condition as one of the main risk factors for suicidal ideation. In addition, we observed that female gender, passive coping, and long working hours during the outbreak increased the risk of developing suicidal thoughts, which is consistent with previous findings during the COVID-19 pandemic [49]. On the contrary, having reliable social support at work and promoting active coping strategies might be critical to preventing suicidal thoughts among HCWs.

Our study found several potential risk factors for HCWs to develop psychological symptoms. However, two factors—using a passive coping style and long working hours during the outbreak—were associated with a higher risk of developing the five evaluated psychological outcomes. Both factors have been previously reported as risk factors for mental health during the pandemic [34, 40, 50]. Regarding long working hours, the participants of this study with at least one mental health problem worked significantly longer daily hours during the outbreak than those who did not show any psychological symptoms. Shortening the shift duration and taking enough time to rest might be critical in avoiding psychological symptoms in HCWs during a major health outbreak. On the other hand, this study revealed that 52.5% of the participants used a passive coping style, contrary to a previous study performed in China that reported active coping styles in 70.2% of the participants [51]. Similarly, studies in the early stages of the COVID-19 pandemic reported that individuals using negative coping styles are more likely to have higher psychological distress [52]. Therefore, psychological interventions at healthcare facilities should include the development of active coping strategies.

Another relevant factor associated with a higher risk of psychological problems was having close contact with confirmed or suspected patients with COVID-19, which increased the risk of depression, anxiety, stress, and PTSD. Similarly, having a chronic disease before the pandemic doubled the risk of psychological distress and suicidal ideation. Previous studies have shown a connection between depression and several chronic diseases [53], which could increase during a major pandemic. Therefore, it is essential to monitor those HCWs having chronic diseases and prioritize their demands in case of future outbreaks.

Likewise, experiencing violence as a healthcare worker during the pandemic increased the risk of manifesting symptoms of depression, anxiety, stress, and PTSD, which have also been reported in previous studies [40, 54]. In this study, 10.6% of the HCWs experienced violence during the pandemic. HCWs have always been at risk of experiencing violence in the workplace, but the pandemic has increased its incidence [55]. This phenomenon, reported in different countries during the COVID-19 pandemic [56, 57], has been highly correlated with mental health problems in HCWs [58]. The lack of medical equipment, medications, ventilators, human resources, and the death of patients have been associated with workplace violence on HCWs [59, 60]. Strict laws against violence on HCWs have been demonstrated to effectively reduce violence at health facilities [60]. Violence at the workplace is preventable, and nationwide actions should be taken to sufficiently protect HCWs.

The female gender was a risk factor for developing depression, anxiety, and suicidal thoughts in the current study. This finding is consistent with several studies carried out in the critical period of the pandemic showing higher rates of psychological distress in women [61, 62]. The results highlight the importance of females’ mental health and the relevance of planning psychological interventions, considering them a higher risk group.

The pandemic has affected the financial condition of many people and organizations around the world due to the restrictions, thus leading to the reduction of work. Although HCWs have increased their workload during this period, our findings demonstrated that 66.9% of the participants reported that the pandemic affected their family’s financial situation. When comparing the different health personnel, nurses showed significantly higher proportions of financial affectation. Additionally, this study found that when the family’s financial situation was affected, the risk of depression and stress increased. These findings are supported by researchers reporting that economic hardship and concerns due to the pandemic could be an essential contributor to anxiety [63] and suicidal ideation during this pandemic and past crises [64]. HCWs included in this study have different incomes and social conditions, more studies are needed to deeply understand the effects of financial stress on the mental health of different health personnel during the pandemic.

In addition, we found that those HCWs who aided Wuhan during the critical period of the pandemic were twice more likely to develop symptoms of depression and anxiety. However, in our sample, only 2.5% of the participants were dispatched to Wuhan. Our study was conducted nationwide, and data were collected from different regions of China. A few participants were from Central China, where Wuhan is located. Although around 42,600 healthcare professionals from all over China were sent to Wuhan to collaborate with local HCWs [65], our sample is only a small fraction of the HCWs in China. Further studies with larger sample sizes and more HCWs who assisted in Wuhan might corroborate our findings.

The fear of infecting relatives was associated with a higher risk of anxiety in this study, and among different health personnel a high proportion of nurses (43.5%) reported to be very worried about this matter. Similar findings in HCWs have been reported in various studies during the pandemic [66]. In addition, the COVID-19-related fears might differ by age groups; younger people has been observed more worried about getting the disease than about spreading it around [13].

Other studies performed during the pandemic have highlighted the importance of access to PPE at the workplace and have shown an association between this factor and psychological distress among HCWs [67]. In this study, 52.6% of the participants considered the PPE at their workplace insufficient or very insufficient. Despite this high proportion, it was not observed as a risk factor in the multivariate analysis. Nevertheless, it is relevant to analyze further and to consider this variable when facing a major pandemic since similar studies performed during the COVID-19 pandemic have reported it as a critical risk factor for mental health [68].

Finally, the results showed that reliable social support at work could be a protective factor against depression, stress, and suicidal ideation, which is consistent with reports that have mentioned the importance of social support and resilience in moderating psychological distress [69]. Moreover, communicating and connecting with colleagues at the workplace, sharing experiences, giving each other advice [70], and teamwork [71] have been reported to improve the mental health of HCWs during the pandemic. Therefore, authorities should implement interventions to enhance work relations and promote peer-to-peer interactions, especially under stressful circumstances.

### Strengths and Limitations of the Study

One of the main strengths of this study was the period when it was performed, 1 year after the initial COVID-19 outbreak. In addition, having a relatively large sample size and a multi-center design could provide a representative sample and a better generalization of the results.

Irrespective of the essential contributions of the current study, it also has some important limitations. Firstly, this study is a cross-sectional design using an online survey through a convenience sampling strategy. This made it difficult to use causal inference and, in this particular case, to calculate the participation rate of the study. In addition, since this study evaluated five psychological outcomes and several pandemic-related factors, some of the psychological scales selected were short but probably not the most accurate, such as the case of suicidal ideation that was evaluated using the last item of the PHQ-9 and not a most widely used scale for this purpose such as Beck scale for suicidal ideation [72, 73]. Another limitation was the impossibility of capturing changes in the mental health of HCWs and its predictors throughout the COVID-19 pandemic. The data for the study was collected during a specific time when the level of the pandemic can be considered as low. Therefore, there is an opportunity to compare our results with similar studies in previous periods, trying to catch the evolution of mental health using aggregated data. The methodology can determine different levels of association of variables, but the causality of risk factors would need a different design. This is why we used a plausible causal model to select potential variables that can impact mental health output, but our results are expressed as possible causation.

### Conclusion

After 1 year of the initial COVID-19 outbreak, Chinese HCWs from different regions manifest a high prevalence of negative psychological impact, including symptoms of depression, stress, anxiety, PTSD, and suicidal ideation. Nurses were more likely to develop symptoms of anxiety and stress when compared with other health personnel. We identified that female gender, passive coping styles, long working hours, close contact with COVID-19 cases, having a chronic disease, experiencing violence as a HCW, having financial stress, fear of infecting relatives, more days spent in isolation, and working in Wuhan during the outbreak are all risk factors for psychological impairment in HCWs.

Our results suggest that developing and promoting programs to improve mental health and identifying those who might need psychological support is still relevant 1 year after the initial outbreak. The programs should be oriented to high-risk groups such as nurses, females, and HCWs having chronic diseases. Our findings led us to conclude that some factors could be modified at the organizational level, such as long working hours, violence at the workplace, financial stress, and reliable social support at work. On the other hand, developing active coping strategies could be critical to improving mental health at the individual level. Furthermore, longitudinal studies are needed to confirm our findings. This study sheds light on future mental health research and provides helpful information to policymakers for this pandemic or future outbreaks.

## Supplementary Material

Supplementary Figure S1 Negative psychological impact calculated based on the scores of three psychological scales (China, 2021).

## References

[1] Xiong J Lipsitz O Nasri F Lui LMW Gill H Phan L Impact of COVID-19 Pandemic on Mental Health in the General Population: A Systematic Review. J Affect Disord (2020) 277:55–64. 10.1016/j.jad.2020.08.001 32799105 PMC7413844

[2] Pappa S Ntella V Giannakas T Giannakoulis VG Papoutsi E Katsaounou P. Prevalence of Depression, Anxiety, and Insomnia Among Healthcare Workers during the COVID-19 Pandemic: A Systematic Review and Meta-Analysis. Brain Behav Immun (2020) 88:901–7. 10.1016/j.bbi.2020.05.026 32437915 PMC7206431

[3] Spoorthy MS Pratapa SK Mahant S. Mental Health Problems Faced by Healthcare Workers Due to the COVID-19 Pandemic-A Review. Asian J Psychiatr (2020) 51:102119. 10.1016/j.ajp.2020.102119 32339895 PMC7175897

[4] United Nations. Policy Brief: Covid-19 and the Need for Action on Mental Health. Policy Br COVID-19 Need Action Ment Heal (2020). p. 1–17.

[5] De Oliveira Souza D. Health of Nursing Professionals: Workload during the COVID-19 Pandemic. Rev Bras Med Trab (2020) 18(4):464–71. 10.47626/1679-4435-2020-600 PMC7934175 33688329

[6] Tiete J Guatteri M Lachaux A Matossian A Hougardy JM Loas G Mental Health Outcomes in Healthcare Workers in COVID-19 and Non-COVID-19 Care Units: A Cross-Sectional Survey in Belgium. Front Psychol (2021) 11:612241. 10.3389/fpsyg.2020.612241 33469439 PMC7813991

[7] Romani M Ashkar K. Burnout Among Physicians. Libyan J Med (2014) 9(1):23556. 10.3402/ljm.v9.23556 PMC3929077 24560380

[8] Atif K Khan HU Ullah MZ Shah FS Latif A. Prevalence of Anxiety and Depression Among Doctors; the Unscreened and Undiagnosed Clientele in Lahore, Pakistan. Pak J Med Sci (2016) 32(2):294–8. 10.12669/pjms.322.8731 27182226 PMC4859009

[9] Salari N Khazaie H Hosseinian-Far A Khaledi-Paveh B Kazeminia M Mohammadi M The Prevalence of Stress, Anxiety and Depression within Front-Line Healthcare Workers Caring for COVID-19 Patients: A Systematic Review and Meta-Regression. Hum Resour Health (2020) 18(1):100. 10.1186/s12960-020-00544-1 33334335 PMC7745176

[10] Carmassi C Foghi C Dell’Oste V Cordone A Bertelloni CA Bui E PTSD Symptoms in Healthcare Workers Facing the Three Coronavirus Outbreaks: What Can We Expect after the COVID-19 Pandemic. Psychiatry Res (2020) 292:113312. 10.1016/j.psychres.2020.113312 32717711 PMC7370915

[11] Jahrami H BaHammam AS AlGahtani H Ebrahim A Faris M AlEid K The Examination of Sleep Quality for Frontline Healthcare Workers during the Outbreak of COVID-19. Sleep Breath (2021) 25(1):503–11. 10.1007/s11325-020-02135-9 32592021 PMC7319604

[12] Mortier P Vilagut G Ferrer M Serra C Molina JD López-Fresneña N Thirty-day Suicidal Thoughts and Behaviors Among Hospital Workers during the First Wave of the Spain COVID-19 Outbreak. Depress Anxiety (2021) 38(5):528–44. 10.1002/da.23129 33393724 PMC8246904

[13] Costanza A Macheret L Folliet A Amerio A Aguglia A Serafini G Covid-19 Related Fears of Patients Admitted to a Psychiatric Emergency Department during and post-lockdown in switzerland: Preliminary Findings to Look Ahead for Tailored Preventive Mental Health Strategies. Medicina (2021) 57(12):1360. 10.3390/medicina57121360 34946305 PMC8707997

[14] Costanza A Amerio A Aguglia A Serafini G Amore M Macchiarulo E From “The Interpersonal Theory of Suicide” to “The Interpersonal Trust”: An Unexpected and Effective Resource to Mitigate Economic Crisis-Related Suicide Risk in Times of Covid-19? Acta Biomed (2021) 92(S6):e2021417. 10.23750/abm.v92iS6.12249 34739460 PMC8851025

[15] Jung H Jung SY Lee MH Kim MS. Assessing the Presence of Post-Traumatic Stress and Turnover Intention Among Nurses Post–Middle East Respiratory Syndrome Outbreak: The Importance of Supervisor Support. Workplace Health Saf (2020) 68(7):337–45. 10.1177/2165079919897693 32146875 PMC7201205

[16] American Psychiatric Association. Diagnostic and Statistical Manual of Mental Disorders. DSM-5. 5th ed (Washington, DC) (2013). p. 947.

[17] Lai J Ma S Wang Y Cai Z Hu J Wei N Factors Associated with Mental Health Outcomes Among Health Care Workers Exposed to Coronavirus Disease 2019. JAMA Netw Open (2020) 3(3):e203976. 10.1001/jamanetworkopen.2020.3976 32202646 PMC7090843

[18] Si MY Su XY Jiang Y Wang WJ Gu XF Ma L Psychological Impact of COVID-19 on Medical Care Workers in China. Infect Dis Poverty (2020) 9(1):113. 10.1186/s40249-020-00724-0 32787929 PMC7422468

[19] Liu N Zhang F Wei C Jia Y Shang Z Sun L Prevalence and Predictors of PTSS during COVID-19 Outbreak in China Hardest-Hit Areas: Gender Differences Matter. Psychiatry Res (2020) 287:112921. 10.1016/j.psychres.2020.112921 32240896 PMC7102622

[20] Zhang H Shi Y Jing P Zhan P Fang Y Wang F. Posttraumatic Stress Disorder Symptoms in Healthcare Workers after the Peak of the COVID-19 Outbreak: A Survey of a Large Tertiary Care Hospital in Wuhan. Psychiatry Res (2020) 294:113541. [Internet]Available from: https://www.sciencedirect.com/science/article/pii/S0165178120332029 10.1016/j.psychres.2020.113541 33128999 PMC7585629

[21] Tan W Hao F McIntyre RS Jiang L Jiang X Zhang L Is Returning to Work during the COVID-19 Pandemic Stressful? A Study on Immediate Mental Health Status and Psychoneuroimmunity Prevention Measures of Chinese Workforce. Brain Behav Immun (2020) 87:84–92. 10.1016/j.bbi.2020.04.055 32335200 PMC7179503

[22] Chen X Arber A Gao J Zhang L Ji M Wang D The Mental Health Status Among Nurses from Low-Risk Areas under Normalized COVID-19 Pandemic Prevention and Control in China: A Cross-Sectional Study. Int J Ment Health Nurs (2021) 30:975–87. 10.1111/inm.12852 33811426 PMC8250992

[23] Wang K Shi H-S Geng F-L Zou L-Q Tan S-P Wang Y Cross-cultural Validation of the Depression Anxiety Stress Scale–21 in China. Psychol Assess (2016) 28(5):e88–100. 10.1037/pas0000207 26619091

[24] Jiang F Liu S Zhao N Xie Y Wang S Ouyang X Psychological Status of the Staff in a General Hospital during the Outbreak of Coronavirus Disease 2019 and its Influential Factors. J Cent South Univ (Medical Sci) (2020) 45(6):641–8. 10.11817/j.issn.1672-7347.2020.200190 32879120

[25] Prins A Bovin MJ Smolenski DJ Marx BP Kimerling R Jenkins-Guarnieri MA The Primary Care PTSD Screen for DSM-5 (PC-PTSD-5): Development and Evaluation within a Veteran Primary Care Sample. J Gen Intern Med (2016) 31(10):1206–11. 10.1007/s11606-016-3703-5 27170304 PMC5023594

[26] Huang R Shen T Ge L Cao L Luo J. Psychometric Properties of the Chinese Version of the Primary Care Post-Traumatic Stress Disorder Screen-5 for Medical Staff Exposed to the COVID-19 Pandemic. Psychol Res Behav Manag (2021) 14:1371–8. 10.2147/PRBM.S329380 34512047 PMC8421668

[27] Costantini L Pasquarella C Odone A Colucci ME Costanza A Serafini G Screening for Depression in Primary Care with Patient Health Questionnaire-9 (PHQ-9): A Systematic Review. J Affect Disord (2021) 279:473–83. 10.1016/j.jad.2020.09.131 33126078

[28] Wang W Bian Q Zhao Y Li X Wang W Du J Reliability and Validity of the Chinese Version of the Patient Health Questionnaire (PHQ-9) in the General Population. Gen Hosp Psychiatry (2014) 36(5):539–44. 10.1016/j.genhosppsych.2014.05.021 25023953

[29] Sahimi HMS Mohd Daud TI Chan LF Shah SA Rahman FHA Nik Jaafar NR. Depression and Suicidal Ideation in a Sample of Malaysian Healthcare Workers: A Preliminary Study during the COVID-19 Pandemic. Front Psychiatry (2021) 12:658174. 10.3389/fpsyt.2021.658174 34025479 PMC8136356

[30] Folkman S Moskowitz JT. Coping: Pitfalls and Promise. Annu Rev Psychol (2004) 55:745–74. 10.1146/annurev.psych.55.090902.141456 14744233

[31] Xie Y. Reliability and Validity of the Simplified Coping Style Questionnaire. Chin J Clin Psychol (1998) 6(2):114–5.

[32] Guo J Feng XL Wang XH van IJzendoorn MH. Coping with COVID-19: Exposure to COVID-19 and Negative Impact on Livelihood Predict Elevated Mental Health Problems in Chinese Adults. Int J Environ Res Public Health (2020) 17(11):E3857. 10.3390/ijerph17113857 32485859 PMC7312167

[33] Chen G Gong J Qi Z Zhong S Su T Wang J The Psychological Status of General Population in Hubei Province during the COVID-19 Outbreak: A Cross-Sectional Survey Study. Front Public Health (2021) 9:203. Available from: https://www.frontiersin.org/article/10.3389/fpubh.2021.622762 . 10.3389/fpubh.2021.622762 PMC8100308 33968877

[34] Yu H Li M Li Z Xiang W Yuan Y Liu Y Coping Style, Social Support and Psychological Distress in the General Chinese Population in the Early Stages of the COVID-19 Epidemic. BMC Psychiatry (2020) 20(1):426. 10.1186/s12888-020-02826-3 32854656 PMC7450895

[35] Lucchini A Giani M Elli S Villa S Rona R Foti G. Nursing Activities Score is Increased in COVID-19 Patients. Intensive Crit Care Nurs (2020) 59:102876. 10.1016/j.iccn.2020.102876 32360493 PMC7177066

[36] Dutheil F Aubert C Pereira B Dambrun M Moustafa F Mermillod M Suicide Among Physicians and Health-Care Workers: A Systematic Review and Meta-Analysis. PLoS One (2019) 14(12):e0226361. 10.1371/journal.pone.0226361 31830138 PMC6907772

[37] Galanis P Vraka I Fragkou D Bilali A Kaitelidou D. Nurses’ Burnout and Associated Risk Factors during the COVID-19 Pandemic: A Systematic Review and Meta-Analysis. J Adv Nurs (2021) 77(8):3286–302. 10.1111/jan.14839 33764561 PMC8250618

[38] Hu D Kong Y Li W Han Q Zhang X Zhu LX Frontline Nurses’ Burnout, Anxiety, Depression, and Fear Statuses and Their Associated Factors during the COVID-19 Outbreak in Wuhan, China: A Large-Scale Cross-Sectional Study. EClinicalMedicine (2020) 24:100424. [Internet]Available from: https://www.sciencedirect.com/science/article/pii/S2589537020301681 . 10.1016/j.eclinm.2020.100424 32766539 PMC7320259

[39] Qiu X Lan Y Miao J Wang H Wang H Wu J A Comparative Study on the Psychological Health of Frontline Health Workers in Wuhan under and after the Lockdown. Front Psychiatry (2021) 12:701032. 10.3389/fpsyt.2021.701032 34234703 PMC8255471

[40] Yang Y Lu L Chen T Ye S Kelifa MO Cao N Healthcare Worker’s Mental Health and Their Associated Predictors during the Epidemic Peak of Covid-19. Psychol Res Behav Manag (2021) 14:221–31. 10.2147/PRBM.S290931 33658870 PMC7918562

[41] Zhang R Lai J Wang Y Huang J Hu S Wang H. Mental Health Outcome and Resilience Among Aiding Wuhan Nurses: One Year after the COVID-19 Outbreak in China. J Affect Disord (2022) 297:348–52. 10.1016/j.jad.2021.10.050 34710499 PMC8564215

[42] Yu X Xu C Wang H Chang R Dong Y Tsamlag L Effective Mitigation Strategy in Early Stage of COVID-19 Pandemic in China. Infect Dis Poverty (2020) 9:141. 10.1186/s40249-020-00759-3 33046120 PMC7549078

[43] Mei S Liang L Ren H Hu Y Qin Z Cao R Association between Perceived Stress and Post-Traumatic Stress Disorder Among Medical Staff during the COVID-19 Epidemic in Wuhan City. Front Public Health (2021) 9:780. 10.3389/fpubh.2021.666460 PMC8356076 34395359

[44] Xiong L-J Zhong B-L Cao X-J Xiong H-G Huang M Ding J Possible Posttraumatic Stress Disorder in Chinese Frontline Healthcare Workers Who Survived COVID-19 6 Months after the COVID-19 Outbreak: Prevalence, Correlates, and Symptoms. Transl Psychiatry (2021) 11(1):374. 10.1038/s41398-021-01503-7 34226510 PMC8256400

[45] Xu X Wang W Chen J Ai M Shi L Wang L Suicidal and Self-Harm Ideation Among Chinese Hospital Staff during the COVID-19 Pandemic: Prevalence and Correlates. Psychiatry Res (2021) 296:113654. 10.1016/j.psychres.2020.113654 33360965 PMC7836678

[46] Smallwood N Willis K. Mental Health Among Healthcare Workers during the COVID-19 Pandemic. Respirology (2021) 26(11):1016–7. 10.1111/resp.14143 34467596 PMC8661789

[47] Gürhan N Beşer NG Polat Ü Koç M. Suicide Risk and Depression in Individuals with Chronic Illness. Community Ment Health J (2019) 55(5):840–8. 10.1007/s10597-019-00388-7 30848413

[48] O’Rourke MC Jamil RT Siddiqui W. Suicide Screening and Prevention. Treasure Island (FL): StatPearls Publishing (2022). 30285348

[49] Jahan I Ullah I Griffiths MD Mamun MA. COVID-19 Suicide and its Causative Factors Among the Healthcare Professionals: Case Study Evidence from Press Reports. Perspect Psychiatr Care (2021) 57(4):1707–11. 10.1111/ppc.12739 33547666 PMC8014758

[50] Khattak SR Saeed I Rehman SU Fayaz M. Impact of Fear of COVID-19 Pandemic on the Mental Health of Nurses in Pakistan. J Loss Trauma (2021) 26(5):421–35. 10.1080/15325024.2020.1814580

[51] Fu W Wang C Zou L Guo Y Lu Z Yan S Psychological Health, Sleep Quality, and Coping Styles to Stress Facing the COVID-19 in Wuhan, China. Transl Psychiatry (2020) 10(1):225. 10.1038/s41398-020-00913-3 32647160 PMC7347261

[52] Wang H Xia Q Xiong Z Li Z Xiang W Yuan Y The Psychological Distress and Coping Styles in the Early Stages of the 2019 Coronavirus Disease (COVID-19) Epidemic in the General mainland Chinese Population: A Web-Based Survey. PLoS One (2020) 15(5):e0233410. 10.1371/journal.pone.0233410 32407409 PMC7224553

[53] Voinov B Richie WD Bailey RK. Depression and Chronic Diseases: it Is Time for a Synergistic Mental Health and Primary Care Approach. Prim Care Companion CNS Disord (2013) 15(2):PCC.12r01468. 10.4088/PCC.12r01468 23930236 PMC3733529

[54] Shi L Wang L Jia X Li Z Mu H Liu X Prevalence and Correlates of Symptoms of post-traumatic Stress Disorder Among Chinese Healthcare Workers Exposed to Physical Violence: a Cross-Sectional Study. BMJ Open (2017) 7(7):e016810. 10.1136/bmjopen-2017-016810 PMC5642665 28765135

[55] Aljohani B Burkholder J Tran QK Chen C Beisenova K Pourmand A. Workplace Violence in the Emergency Department: a Systematic Review and Meta-Analysis. Public Health (2021) 196:186–97. [Internet]Available from: https://www.sciencedirect.com/science/article/pii/S0033350621000676 . 10.1016/j.puhe.2021.02.009 34246105

[56] Ghareeb NS El-Shafei DA Eladl AM. Workplace Violence Among Healthcare Workers during COVID-19 Pandemic in a Jordanian Governmental Hospital: the Tip of the Iceberg. Environ Sci Pollut Res (2021) 28:61441–9. 10.1007/s11356-021-15112-w PMC8233595 34173953

[57] Bhatti OA Rauf H Aziz N Martins RS Khan JA. Violence against Healthcare Workers during the COVID-19 Pandemic: A Review of Incidents from a Lower-Middle-Income Country. Ann Glob Health (2021) 87(1):41. 10.5334/aogh.3203 33977084 PMC8064297

[58] Wang W Lu L Kelifa MM Yu Y He A Cao N Mental Health Problems in Chinese Healthcare Workers Exposed to Workplace Violence during the Covid-19 Outbreak: A Cross-Sectional Study Using Propensity Score Matching Analysis. Risk Manag Healthc Pol (2020) 13:2827–33. 10.2147/RMHP.S279170 PMC7721299 33299370

[59] Muñoz Del Carpio-Toia A Begazo Muñoz Del Carpio L Mayta-Tristan P Alarcón-Yaquetto DE Málaga G. Workplace Violence against Physicians Treating COVID-19 Patients in Peru: A Cross-Sectional Study. Jt Comm J Qual Patient Saf (2021) 47(10):637–45. Available from: https://pubmed.ncbi.nlm.nih.gov/34257040 . 10.1016/j.jcjq.2021.06.002 34257040 PMC8200256

[60] Manoj MA Padubidri JR Saran J Rao SJ Shetty BSK D’Souza H. Violence against Healthcare Personnel in India: Covid-19 Prompts Stricter Laws. Med Leg J (2021) 89(4):260–3. 10.1177/00258172211006276 34013804

[61] Liu S Yang L Zhang C Xu Y Cai L Ma S Gender Differences in Mental Health Problems of Healthcare Workers during the Coronavirus Disease 2019 Outbreak. J Psychiatr Res (2021) 137:393–400. 10.1016/j.jpsychires.2021.03.014 33765451 PMC7962932

[62] Hao Q Wang D Xie M Tang Y Dou Y Zhu L Prevalence and Risk Factors of Mental Health Problems Among Healthcare Workers during the COVID-19 Pandemic: A Systematic Review and Meta-Analysis. Front Psychiatry (2021) 12:567381. [Internet]Available from: https://pubmed.ncbi.nlm.nih.gov/34211406 . 10.3389/fpsyt.2021.567381 34211406 PMC8239157

[63] Wilson JM Lee J Fitzgerald HN Oosterhoff B Sevi B Shook NJ. Job Insecurity and Financial Concern during the COVID-19 Pandemic Are Associated with Worse Mental Health. J Occup Environ Med (2020) 62(9):686–91. 10.1097/JOM.0000000000001962 32890205

[64] Gunnell D Appleby L Arensman E Hawton K John A Kapur N Suicide Risk and Prevention during the COVID-19 Pandemic. The lancet Psychiatry (2020) 7(6):468–71. 10.1016/s2215-0366(20)30171-1 32330430 PMC7173821

[65] Ning Y Ren R Nkengurutse G. China’s Model to Combat the COVID-19 Epidemic: a Public Health Emergency Governance Approach. Glob Health Res Policy (2020) 5(1):34. 10.1186/s41256-020-00161-4 32685691 PMC7358318

[66] De Kock JH Latham HA Leslie SJ Grindle M Munoz SA Ellis L A Rapid Review of the Impact of COVID-19 on the Mental Health of Healthcare Workers: Implications for Supporting Psychological Well-Being. BMC Public Health (2021) 21(1):104. 10.1186/s12889-020-10070-3 33422039 PMC7794640

[67] Mediavilla R Fernández-Jiménez E Martínez-Alés G Moreno-Küstner B Martínez-Morata I Jaramillo F Role of Access to Personal Protective Equipment, Treatment Prioritization Decisions, and Changes in Job Functions on Health Workers’ Mental Health Outcomes during the Initial Outbreak of the COVID-19 Pandemic. J Affect Disord (2021) 295:405–9. [Internet]Available from: https://www.sciencedirect.com/science/article/pii/S0165032721008648 . 10.1016/j.jad.2021.08.059 34507219 PMC8403068

[68] Sampaio F Sequeira C Teixeira L. Nurses’ Mental Health during the Covid-19 Outbreak: A Cross-Sectional Study. J Occup Environ Med (2020) 62(10):783–7. 10.1097/JOM.0000000000001987 32769803

[69] Leng M Wei L Shi X Cao G Wei Y Xu H Mental Distress and Influencing Factors in Nurses Caring for Patients with COVID-19. Nurs Crit Care (2021) 26(2):94–101. 10.1111/nicc.12528 33448567

[70] Kotera Y Ozaki A Miyatake H Tsunetoshi C Nishikawa Y Kosaka M Qualitative Investigation into the Mental Health of Healthcare Workers in Japan during the COVID-19 Pandemic. Int J Environ Res Public Health (2022) 19(1):568. 10.3390/ijerph19010568 35010828 PMC8744919

[71] Rose S Hartnett J Pillai S. Healthcare Worker’s Emotions, Perceived Stressors and Coping Mechanisms during the COVID-19 Pandemic. PLoS One (2021) 16(7):e0254252. 10.1371/journal.pone.0254252 34242361 PMC8270181

[72] Baertschi M Costanza A Canuto A Weber K. The Dimensionality of Suicidal Ideation and its Clinical Implications. Int J Methods Psychiatr Res (2019) 28(1):e1755. 10.1002/mpr.1755 30426604 PMC6877148

[73] Beck AT Kovacs M Weissman A. Assessment of Suicidal Intention: the Scale for Suicide Ideation. J Consult Clin Psychol (1979) 47(2):343–52. 10.1037//0022-006x.47.2.343 469082

