# Supplementary figures and images for: A Multi-Center Study on the Negative Psychological Impact and Associated Factors in Chinese Healthcare Workers 1 Year After the COVID-19 Initial Outbreak
Source: Int J Public Health. 2022 Aug 25;67:1604979. doi: 10.3389/ijph.2022.1604979 (PMC9454095; doi:10.3389/ijph.2022.1604979)

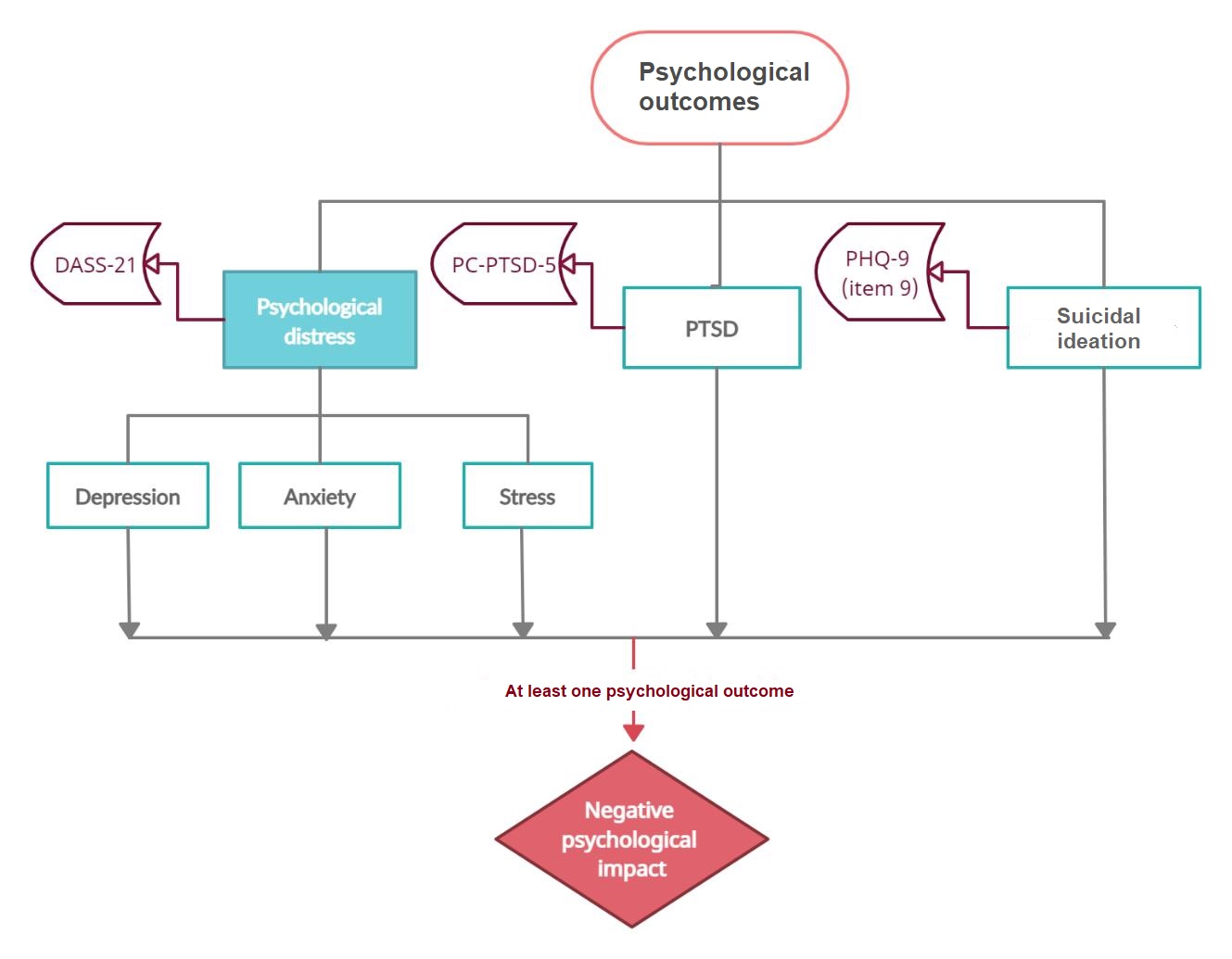

Supplement: Supplementary file 1 [file Image1.jpeg]
